# Exploring the Risks, Benefits, Advances, and Challenges in Internet Integration in Medicine With the Advent of 5G Technology: A Comprehensive Review

**DOI:** 10.7759/cureus.48767

**Published:** 2023-11-13

**Authors:** Varun Agrawal, Suyash Agrawal, Aarya Bomanwar, Tanishq Dubey, Arpita Jaiswal

**Affiliations:** 1 Medicine, Jawaharlal Nehru Medical College, Datta Meghe Institute of Higher Education and Research, Wardha, IND; 2 Obstetrics and Gynaecology, Jawaharlal Nehru Medical College, Datta Meghe Institute of Higher Education and Research, Wardha, IND

**Keywords:** ethical standards, precision medicine, cybersecurity, telemedicine, healthcare integration, 5g technology

## Abstract

The integration of 5G technology in the healthcare sector is poised to bring about transformative changes, offering numerous advantages such as enhanced telemedicine services, expedited data transfer for medical records, improved remote surgery capabilities, real-time monitoring and diagnostics, advancements in wearable medical devices, and the potential for precision medicine. However, this technological shift is not without its concerns, including potential health implications related to 5G radiation exposure, heightened cybersecurity risks for medical devices and data systems, potential system failures due to technology dependence, and privacy issues linked to data breaches in healthcare. We are striking a balance between harnessing these benefits and addressing the associated risks. Achieving this equilibrium requires the establishment of a robust regulatory framework, ongoing research into the health impacts of 5G radiation, the implementation of stringent cybersecurity measures, education and training for healthcare professionals, and the development of ethical standards. The future of 5G in the medical field holds immense promise, but success depends on our ability to navigate this evolving landscape while prioritizing patient safety, privacy, and ethical practice.

## Introduction and background

The fifth generation of wireless technology, commonly known as 5G, represents a significant leap forward in telecommunications. It builds upon its predecessors, 2G, 3G, and 4G, by offering faster data transfer speeds, lower latency, and increased network capacity. It employs a combination of new infrastructure, advanced radio frequencies, and innovative communication protocols to enable high-speed data transmission, opening up new possibilities across various sectors, including healthcare [1].

The healthcare industry stands to benefit significantly from the integration of 5G technology. With its promise of lightning-fast data exchange and minimal latency, 5G has the potential to revolutionize medical practices, enhance patient care, and drive innovation in the field. This significance extends to various medical applications, such as telemedicine, remote surgery, real-time monitoring, and more, ultimately leading to improved patient outcomes and healthcare services [2].

This comprehensive review explores the manifold advantages and potential risks of incorporating 5G technology in the medical sector. As 5G continues to gain prominence and permeate various aspects of healthcare, it is crucial to assess the opportunities it presents, as well as the challenges it may introduce. By examining the latest research, studies, and developments, this review seeks to provide a well-rounded perspective on the role of 5G in medicine, enabling healthcare professionals, policymakers, and the general public to make informed decisions and shape the future of healthcare in the 5G era.

## Review

Benefits of 5G technology in medicine

Enhanced Telemedicine Services

High-definition video conferencing: This technology ensures that video conferences between healthcare professionals and patients are of the highest quality. The crystal-clear video feed facilitates a comprehensive visual examination, allowing doctors to observe subtle details like skin conditions, wounds, or symptoms. This level of clarity is especially invaluable when diagnosing conditions that require visual analysis [3].

Real-time data transmission: With minimal latency and high data transfer speeds, 5G enables real-time data transmission, ensuring that medical data, including vital signs and patient history, can be communicated instantaneously. This capability is pivotal in situations where timely decisions and diagnoses are critical [4].

Remote expertise access: Patients in remote or underserved areas benefit significantly from 5G-enabled telemedicine. They can virtually connect with medical experts regardless of geographical constraints. Access to expert medical advice reduces the need for extensive travel and physical consultations, ultimately improving healthcare accessibility and reducing the burden on healthcare infrastructure [5].

Accelerated Data Transfer for Medical Records

Efficient data exchange for swift communication: Its high-speed data transfer capabilities are a game-changer in healthcare, ensuring the swift exchange of medical records, including patient history, test results, and images. This efficiency streamlines the communication of critical patient information among healthcare professionals, reducing delays in diagnosis and treatment. For instance, when a patient is transferred between healthcare facilities or is seeking a second opinion from a specialist in another location, 5G technology ensures that all relevant medical data is quickly accessible, contributing to faster and more accurate decision-making [6].

Enhanced collaboration and comprehensive care: This technology empowers healthcare professionals across various locations to access patient data in real time. This capability fosters collaboration among specialists, allowing them to work together on complex cases and providing a more comprehensive view of a patient's medical history. In multi-disciplinary healthcare settings, such as cancer treatment teams or trauma centers, this can significantly improve the quality of care. Specialists can simultaneously review patient records, diagnostic images, and test results, leading to well-informed treatment decisions and improved patient outcomes [7].

Timely decision-making in critical situations: In emergencies, where every second is crucial, 5G's rapid data transfer capabilities are precious. Whether in the emergency room, during remote consultations, or when responding to emergencies in the field, quick access to medical records and imaging is vital for making time-sensitive decisions. For example, in a trauma situation, rapid access to a patient's medical history, allergies, and previous treatments can be lifesaving. Similarly, for telemedicine consultations during emergencies, such as stroke or heart attack, 5G technology ensures that specialists can promptly assess the situation and provide timely intervention guidance, potentially saving lives [8].

Data security and privacy compliance: While enabling fast data transfer, 5G ensures these exchanges are secure and compliant with stringent privacy regulations. Robust encryption and authentication protocols protect patient data during transit, maintaining patient confidentiality and integrity. This is vital to build and maintain patient trust in healthcare systems, as patients need assurance that their sensitive medical information is safeguarded, and privacy is upheld. Compliance with privacy regulations such as the Health Insurance Portability and Accountability Act (HIPAA) in the United States is imperative, and 5G technology is equipped to meet these requirements, helping to prevent data breaches and unauthorized access [9].

Improved Remote Surgery Capabilities

Low latency for precise control: One of the defining features of 5G technology in telesurgery is its minimal latency, which ensures that surgical commands and actions are transmitted in near real time. This low delay is crucial for the success of telesurgery, as it enables precise, instantaneous control of surgical instruments. Surgeons can remotely manipulate instruments with remarkable accuracy, simulating in-person surgery even when physically separated from the patient by great distances. This level of precision is vital for delicate and complex surgical procedures, as it minimizes the risk of unintended movements or delays in response, contributing to the safety and success of the surgery [10].

Extending expertise to underserved areas: Telesurgery powered by 5G technology significantly extends the reach of expert surgical care. Surgeons can perform procedures on patients in remote or underserved areas where access to specialized medical expertise is often limited. This democratization of surgical care addresses geographic disparities in healthcare and enhances patient access to skilled surgeons. Patients who would otherwise face barriers to receiving specialized surgical treatment can now benefit from the expertise of leading surgeons, regardless of their location [11].

Life-saving potential in emergencies: Telesurgery has substantial life-saving potential, particularly in emergencies. For example, in cases of accidents, natural disasters, or critical medical emergencies, remote surgery can provide immediate access to essential surgical interventions. Even in areas without fully equipped hospitals or surgical specialists, telesurgery can connect remote surgeons with patients needing urgent care. This capability can be the difference between life and death, offering rapid, expert medical intervention when time is of the essence [12].

Reducing travel risks and burdens: Traditionally, patients needing specialized surgical care often had to undertake long and sometimes risky journeys to reach surgical centers, which could be located far from their homes. With telesurgery and 5G technology, the need for extensive travel is minimized. Patients can receive high-quality surgical care without having to travel great distances, reducing the associated risks and burdens on both patients and their families. This is particularly valuable for individuals with limited mobility, those living in remote areas, or patients requiring frequent follow-up procedures, as it enhances the accessibility and convenience of surgical care [10].

Facilitation of Real-Time Monitoring and Diagnostics

Continuous data transmission: This technology's remarkable data transfer speeds and minimal latency provide the capability for the continuous and uninterrupted transmission of patient data. This means vital signs, health parameters, and other relevant information can be sent to healthcare providers in real time without interruptions or delays. As a result, healthcare professionals have immediate access to the most current and up-to-date patient data, allowing them to make informed decisions based on the patient's real-time condition [13].

Timely intervention: Real-time monitoring is instrumental in enabling healthcare professionals to intervene promptly in case of any concerning changes in a patient's condition. This is especially crucial for patients with chronic illnesses, such as diabetes, heart disease, or respiratory conditions. With real-time data, healthcare providers can detect subtle deviations from a patient's baseline health parameters and respond quickly to prevent potential complications or exacerbations of the condition. Timely interventions can include medication adjustments, lifestyle recommendations, or even hospital admissions when necessary, reducing the risk of severe health episodes and improving overall patient outcomes [14].

Personalized care: The ability to monitor patients in real-time supports delivering highly personalized care plans. Healthcare providers can tailor treatments, interventions, and recommendations based on the most recent and accurate patient data. This personalization ensures that each patient receives care uniquely suited to their current health status, needs, and goals. Whether it involves adjusting medication dosages, dietary recommendations, or exercise regimens, real-time monitoring allows for dynamic and individualized care, enhancing the patient's overall experience and health outcomes [15].

Early detection: Real-time monitoring is particularly valuable for the early detection of health issues. It enables the identification of subtle changes in a patient's health status, often before noticeable symptoms manifest. This early detection can lead to quicker diagnoses and more successful treatment outcomes. For example, in cases of conditions like cancer or cardiovascular diseases, real-time monitoring can reveal irregularities in physiological parameters or biomarkers at an early stage, facilitating early intervention and treatment. Early detection can result in more manageable and curable health conditions, ultimately improving patient prognosis and quality of life [16].

Advancements in Wearable Medical Devices

Sophisticated health monitoring: This technology empowers wearable medical devices to conduct highly sophisticated and accurate health monitoring. These devices can track a broader array of health parameters, going beyond basic metrics like heart rate and step count. With 5G, wearables can monitor and transmit data related to complex health indicators such as sleep patterns, blood glucose levels, ECG data, and more, all in real time. This heightened sophistication provides a more comprehensive understanding of an individual's health, enabling early detection and proactive management of health issues [17].

Real-time data sharing: The low latency and high data transfer speeds of 5G networks enable instantaneous data sharing between wearable devices and healthcare providers. This means that patients can share their health data with their physicians or healthcare teams in real time, regardless of geographical distances. This real-time data sharing fosters a more proactive approach to healthcare management. Healthcare professionals can monitor patients remotely and provide timely interventions or guidance based on the real-time data received, even when patients are at home or in remote locations [3].

Proactive healthcare: With access to real-time health data, patients and healthcare providers can take proactive steps to manage health and promptly address potential issues. For example, changes in vital signs, anomalies in ECG readings, or deviations from established health parameters can trigger immediate alerts or interventions. This enhances the timeliness and effectiveness of healthcare, potentially preventing the exacerbation of medical conditions or complications. Proactive healthcare management is precious for individuals with chronic illnesses, as it allows for early intervention and adjustment of treatment plans [18].

Improved overall wellness: The continuous health monitoring made possible by 5G-enabled wearables extends beyond managing chronic conditions. It promotes overall wellness by encouraging individuals to make healthier choices and adopt more active lifestyles. With real-time awareness of their health data, individuals can set and achieve wellness goals, such as maintaining a healthy heart rate, getting sufficient sleep, or managing stress levels. This proactive approach to wellness empowers individuals to take ownership of their health, make informed lifestyle decisions, and prevent the onset of chronic diseases, contributing to an improved quality of life [19].

Unlocking the Potential of 5G in Genomic Healthcare

Harnessing the power of 5G technology in precision medicine holds immense promise for revolutionizing patient care. One of the key advantages lies in the ability of 5G to facilitate real-time genomic analysis, a pivotal component of personalized treatments.

Real-time genomic analysis: The high-speed data transfer capabilities of 5G are instrumental in enabling the swift and accurate analysis of extensive genomic datasets. In the context of decoding a patient's genetic information, 5G accelerates the data-intensive process of genomic analysis. By swiftly transmitting the data generated by genomic sequencing machines to high-performance computing systems, 5G facilitates real-time interpretation. This rapid analysis of a patient's genome represents a significant leap forward, promising profound implications for healthcare advancement [20].

Tailored treatment plans: Access to a patient's genetic profile empowers healthcare professionals to craft tailored treatment plans. These plans specifically target the genetic factors contributing to an individual's condition. By customizing treatments based on genetic makeup, healthcare providers enhance effectiveness and mitigate the likelihood of adverse effects. Tailored treatment plans maximize positive outcomes, minimizing the burden of ineffective or harmful interventions [21].

Early disease detection: Genomic data analysis, facilitated by 5G, has the potential to identify genetic predispositions to diseases before symptoms manifest. This proactive approach is a cornerstone of precision medicine. Understanding a patient's genetic risk factors enables healthcare professionals to implement preventative measures crucial for diseases like certain cancers or hereditary disorders. Timely intervention, made possible by early detection, can be lifesaving [22].

Optimized medication selection: Knowledge of a patient's genetic makeup guides healthcare professionals in selecting the most appropriate medications. This personalized approach minimizes the need for trial and error, reducing the risk of prescribing ineffective or adversely reacting medications. Optimized medication selection aligns with patient-centered care, improving outcomes and reducing healthcare costs associated with unsuccessful treatments and complications [23].

Enhanced patient outcomes: Embracing precision medicine and personalized treatments significantly elevates patient outcomes. Healthcare becomes inherently patient-centric, focusing on individual needs and genetic characteristics. This approach increases the likelihood of treatment success, fostering a better quality of life and nurturing increased trust in the healthcare system among patients [21].

Risks associated with 5G technology in medicine

Potential Health Concerns of 5G Radiation Exposure

RF radiation exposure: One of the primary concerns surrounding the deployment of 5G technology is the potential health impact of increased radiofrequency (RF) radiation exposure. Unlike previous generations of wireless technology, 5G employs higher-frequency millimeter waves for data transmission. These waves fall within the RF spectrum, and there is apprehension about the increased exposure to RF radiation due to the densification of 5G infrastructure. The concern is that the closer spacing of 5G infrastructure, such as small cells and antennas, may result in higher RF radiation levels near these devices. This issue is particularly relevant in healthcare facilities where 5G infrastructure may be densely deployed to support medical applications [24].

Health impact debate: The scientific community has engaged in a debate regarding the health effects of 5G radiation. Various studies and research have yielded mixed findings, leading to differing conclusions. While some studies suggest potential risks associated with RF radiation exposure, others have not found conclusive evidence of harm. These disparities in research findings have raised concerns and underlined the need for further investigation. It is essential to consider the methodological variations, exposure conditions, and study designs that contribute to the discrepancies in research outcomes [25].

Long-term effects: Understanding the long-term health implications of increased RF radiation exposure from 5G technology is particularly challenging. The deployment of 5G is relatively recent, and long-term studies assessing the cumulative effects of prolonged exposure to 5G radiation are limited. Prolonged and cumulative exposure may present different health risks than short-term exposure. Therefore, the potential for health effects over years or decades must be considered and further studied [26].

Monitoring and research: To address these concerns, ongoing monitoring and research are essential. Scientific investigations should continue to assess the potential health impacts of 5G technology, especially in healthcare settings where exposure may be more prolonged or concentrated. Longitudinal studies that track individuals' health outcomes over extended periods of 5G exposure are vital for gaining insights into latent health risks. Additionally, research should focus on specific medical applications of 5G technology to evaluate exposure levels and potential health consequences in healthcare environments [27].

Regulatory oversight: Regulatory bodies and public health organizations must proactively establish safety standards and guidelines for RF radiation exposure, particularly in healthcare facilities. These standards should be regularly updated to reflect the latest scientific findings. Regulatory oversight is crucial to ensure that healthcare institutions and technology providers adhere to safety measures and best practices that protect patients and healthcare workers from potential health risks associated with 5G radiation. Public health organizations should collaborate with experts and researchers to set evidence-based exposure limits and ensure that 5G technology is deployed safely in healthcare settings. This collaboration helps address the ongoing debate and uncertainty about 5G radiation's health effects while prioritizing public safety and healthcare quality [28].

Cybersecurity Vulnerabilities in Medical Devices

Rising connectivity in the era of 5G: With the advent of 5G technology, the interconnectivity of medical devices in healthcare settings has reached unprecedented levels. This enhances the speed and efficiency of communication and significantly expands the attack surface for potential cyber threats [29]. From infusion pumps to imaging equipment, the spectrum of devices susceptible to exploitation by malicious actors seeking unauthorized access widens as they become more interconnected. The unique implications of 5G on these vulnerabilities merit specific consideration.

Data breaches and the 5G paradigm: In the context of 5G, cyberattacks on medical devices can lead to data breaches, exposing sensitive patient information to unprecedented risks. Data transmission speed and volume in 5G networks amplify the potential impact of breaches, posing elevated risks to patient records, medical histories, and personal identification data. This compromises patient privacy and escalates legal and ethical concerns to new heights [30].

Disruption of care in the 5G age: In the era of 5G, cybersecurity vulnerabilities transcend mere data compromise; they can disrupt medical procedures and treatments. The heightened connectivity facilitated by 5G networks increases the susceptibility of devices to interference by malicious actors, potentially jeopardizing patient safety and the quality of care. Tampering with infusion pumps or altering dosages through 5G-enabled avenues could lead to severe consequences, even life-threatening situations [30].

The integrity of healthcare systems in the 5G landscape: Attacks on interconnected medical devices, particularly in the 5G landscape, pose a critical threat to the integrity of healthcare systems. The increased speed and efficiency of 5G networks make them more susceptible to system failures, potentially affecting the availability and reliability of critical healthcare services. Downtime or disruption in 5G-enabled healthcare systems can have cascading effects on patient care and clinical operations, magnifying the potential harm [31].

Patient safety challenges in the 5G-connected healthcare environment: The cybersecurity of medical devices in the context of 5G directly impacts patient safety. Breaches, tampering, or unauthorized access can now have even more immediate and profound consequences, particularly when real-time medical interventions are necessary. Patient safety, a fundamental aspect of care, faces heightened challenges in the 5G-connected healthcare environment [32].

Safeguarding measures in 5G-enabled medical technology: In embracing 5G-enabled medical technology, the healthcare industry must prioritize robust cybersecurity measures. Encryption for protecting data at rest and in transit, stringent access controls, regular software updates, and advanced intrusion detection systems become even more critical in the 5G era. Integrating these measures into the design, development, and maintenance of medical devices and healthcare networks is imperative to mitigate the unique risks posed by 5G [31].

Regulatory compliance in the 5G landscape: In the age of 5G, adherence to regulatory standards for cybersecurity in medical technology becomes paramount. Compliance with regulations such as HIPAA in the United States is not just a legal requirement but a crucial aspect of maintaining patient trust and safety. The dynamic nature of 5G networks necessitates ongoing adjustments to these compliance standards to address emerging challenges [33].

Dependence on Technology and Potential System Failures

Overreliance on 5G: As healthcare systems integrate 5G technology, there is a growing dependency on its seamless functioning. The reliance on 5G networks for critical medical services, data exchange, and real-time monitoring raises concerns about system fragility. Overreliance on any single technology can create vulnerabilities, making healthcare systems more susceptible to disruptions if 5G services encounter problems [2].

Network outages: Like any technology, 5G networks can experience outages due to technical issues or other unforeseen circumstances. Even short periods of network unavailability can have severe consequences for patient care and medical operations. Network outages can disrupt the transmission of critical patient data, hinder communication between healthcare providers, and impede access to medical records, potentially affecting the quality and timeliness of care [34].

Technical glitches: Technical glitches within the 5G infrastructure or associated medical devices can disrupt the flow of information and services. These disruptions can impact diagnostic procedures, treatment plans, and patient monitoring. Technical glitches may lead to delays in patient care, misinterpretation of data, or even errors in medical equipment operations, highlighting the need for robust systems and backup measures to ensure continuity of care [3].

Cyberattacks: The dependence on interconnected systems also exposes healthcare to the risk of cyberattacks. Malicious actors may target 5G-enabled healthcare technology to compromise the availability and integrity of critical medical services. Cyberattacks can result in system downtime, data breaches, and the manipulation of medical information, potentially endangering patient safety and privacy [35].

Patient safety: Any disruption to healthcare services can directly impact patient safety. Delays in diagnosis, treatment, or monitoring can have life-threatening consequences, particularly in emergencies. Patients' health and well-being depend on timely and accurate access to medical services and information. Disruptions in these services can result in adverse outcomes and compromise patient safety [36].

Mitigation strategies: To address these risks, healthcare systems must implement robust mitigation strategies. This includes developing backup systems, redundancy in critical processes, and contingency plans to maintain service continuity during 5G-related issues. These strategies should be designed to minimize disruptions and ensure that patients continue to receive care, even in the face of network outages or technical glitches [37].

Collaboration and preparedness: Collaboration among healthcare providers, technology vendors, and regulatory bodies is essential to ensure preparedness for potential system failures. Regular testing and drills can help healthcare professionals respond effectively to disruptions. Preparedness measures should encompass technical aspects, such as redundant systems and the human factor, including training healthcare staff to handle unexpected situations and maintain patient safety during 5G-related issues. Continuous communication and coordination are vital to addressing these risks and ensuring the resilience of healthcare systems [38].

Privacy Issues and Data Breaches in Healthcare

Sensitivity of medical data: Healthcare data, encompassing patient records, diagnostic information, treatment history, and medication lists, is highly sensitive and confidential. Leakage of this information poses serious risks, including identity theft, insurance fraud, and potential discrimination. Safeguarding the sensitivity of medical data is crucial to uphold patient trust in healthcare services [39].

Data breaches and the impact of 5G: The integration of 5G technology expands the potential attack surface for cybercriminals targeting healthcare systems. While the vulnerability of medical data is inherent in the internet era, the adoption of 5G increases the likelihood of data breaches. Unauthorized access can result in severe consequences such as financial losses, legal liabilities, damage to the reputation of healthcare organizations, and, most importantly, compromise of patient privacy. The prevention of data breaches becomes even more imperative in the context of 5G technology [40].

Legal and ethical obligations: Healthcare organizations are obligated to adhere to legal and ethical standards for protecting patient data. Compliance with data protection regulations, including HIPAA in the United States, is imperative. Failure to meet these obligations can lead to severe legal repercussions, including fines and legal actions. Ethical obligations underscore the fundamental responsibility of healthcare organizations to uphold patient privacy and trust [41].

Building and maintaining patient trust: Privacy violations and data breaches significantly undermine patient trust in healthcare services. Patients must have confidence that their sensitive information is secure and confidential. Fostering and maintaining this trust is essential for effective healthcare delivery. Patients are more likely to share accurate and complete information with healthcare providers when they trust the security of their data. Trust forms the foundation of the patient-provider relationship and is integral to successful healthcare outcomes [42].

Security Measures

Encryption and access controls: To protect medical data, robust encryption methods should be employed for both data in transit and at rest, ensuring confidentiality and integrity. Access controls and authentication measures are equally important, limiting access to authorized personnel only. These security measures collectively help safeguard patient data from unauthorized access and potential breaches [43].

Ensuring secure data storage: Practices such as regular data backups and the implementation of disaster recovery plans are essential in healthcare. Data should be stored securely to ensure its availability even in the face of unexpected events like hardware failures, natural disasters, or cyberattacks. The ability to recover and restore data is crucial for maintaining the continuity of patient care and preserving medical records [44].

Continuous monitoring for data security: It is vital for maintaining data security that data systems be continuously monitored for potential vulnerabilities and unauthorized access. Intrusion detection systems, capable of detecting and responding to security incidents in real time, play a crucial role in ensuring ongoing data security. Continuous monitoring allows for proactive responses to threats, reducing the impact of potential breaches and ensuring healthcare organizations remain vigilant in safeguarding patient information [45].

Current research and findings on 5G technology in medicine

Cybersecurity Measures for Medical Devices and Data Systems

Vulnerability assessments: Researchers are actively conducting comprehensive assessments of existing healthcare technologies, particularly those integrated with 5G networks. These assessments involve identifying and evaluating weaknesses and potential entry points for cyber threats. Healthcare organizations can take proactive measures to mitigate risks and strengthen their cybersecurity posture by understanding the vulnerabilities. Vulnerability assessments are a critical first step in ensuring the security of patient data and healthcare systems [37].

Advanced security protocols: Developing advanced security protocols is a paramount focus of cybersecurity research in healthcare. These protocols protect patient data and healthcare systems from cyber threats. They take into account the unique requirements of the healthcare sector. They may involve the creation of secure communication standards and encryption techniques specifically tailored to the intricacies of healthcare data exchange. Advanced security protocols are essential for safeguarding sensitive medical information and ensuring the integrity of healthcare operations [46].

Intrusion detection systems: Intrusion detection systems are being developed to monitor and swiftly detect potential security breaches in real time. These systems can identify unauthorized access, unusual activities, or deviations from established patterns within medical devices and data systems. By providing real-time alerts, intrusion detection systems enable rapid response and remediation, reducing the impact of security incidents and protecting patient data [47].

Encryption methods: Encryption is a fundamental pillar of healthcare cybersecurity. Researchers are exploring innovative encryption methods to protect patient data during storage and transmission. These methods ensure that even if data is compromised, it remains unreadable to unauthorized parties. Encryption is a vital safeguard against data breaches and unauthorized access, helping to maintain the confidentiality and integrity of patient records and sensitive medical information [48].

Regulatory compliance: Compliance with regulatory standards and guidelines for medical device cybersecurity is a critical aspect of healthcare cybersecurity research. Researchers, in collaboration with healthcare providers and device manufacturers, work to ensure that new and existing technologies adhere to these standards. Compliance with regulations, such as those outlined in HIPAA, is essential for maintaining the integrity of healthcare systems and protecting patient privacy. Regulatory compliance sets a baseline for cybersecurity practices in healthcare [40].

Interdisciplinary collaboration: Cybersecurity in healthcare often demands collaboration among cybersecurity experts, healthcare professionals, device manufacturers, and regulatory bodies. This interdisciplinary approach fosters comprehensive and practical solutions to protect patient data and medical devices. By bringing together diverse expertise, healthcare organizations can develop holistic strategies to address cybersecurity challenges, share best practices, and ensure a coordinated response to emerging threats [49].

Education and training: Healthcare professionals receive education and training on cybersecurity best practices. This includes teaching them how to identify and respond to potential threats, as well as how to adhere to secure data handling and access control practices. Education and training are vital components in creating a healthcare workforce that is proactive in safeguarding patient data and maintaining the security of medical devices and systems. Healthcare professionals play a crucial role in the cybersecurity strategy by being vigilant and informed about potential risks [50].

Integration of 5G Technology in Clinical Trials and Research

Remote patient monitoring: The capacity of 5G technology for remote patient monitoring revolutionizes clinical trials by enabling researchers to collect data from participants with unprecedented precision and real-time transmission. In clinical trials, this capability is precious as it eliminates the need for frequent in-person visits, reducing participant burden and improving the overall trial experience. It allows for continuous monitoring of vital signs, symptoms, and treatment responses, providing researchers with a wealth of data on participants' health status [51].

Real-time data collection: The low latency of 5G networks ensures that data collected during clinical trials can be transmitted and analyzed in real time. This instantaneous data analysis can transform the research process by enabling researchers to identify trends and insights as they occur. It minimizes delays between data collection and analysis, allowing researchers to adapt their research protocols or treatment plans promptly. Real-time data collection also enhances the safety of clinical trials by enabling rapid detection of adverse events or treatment inefficacies [52].

Collaborative research: The high data transfer speeds of 5G support collaborative research efforts globally. Researchers from different locations can seamlessly share data, collaborate on projects, and engage in teleconferences with minimal latency. This promotes a collaborative and interdisciplinary approach to medical research, allowing experts from various fields and geographical locations to work together efficiently. Collaborative research leverages the collective expertise of the global scientific community, potentially accelerating breakthroughs in medical science [3].

Accelerated research: With the data transfer capabilities of 5G, medical researchers can access and analyze vast datasets more rapidly than ever before. This acceleration in data processing has the potential to expedite the research process, leading to quicker research outcomes. It may accelerate the development of new medical treatments, technologies, and interventions by enabling researchers to reach conclusions and make evidence-based decisions in a shorter time frame. This is particularly vital in clinical trials where time is a critical factor [53].

Improved patient recruitment: This technology simplifies recruiting participants for clinical trials. Researchers can cast a wider net and reach a more diverse and geographically distributed patient population. This broadened reach enhances the generalizability of research findings and ensures that the results apply to a more representative population. It also helps expedite the patient recruitment process, often a bottleneck in clinical trial timelines [54].

Enhanced data security: While data sharing and collaboration are facilitated by 5G technology, robust security measures are equally critical. Researchers are actively exploring cybersecurity protocols that protect the privacy of research participants and maintain data integrity. This includes implementing robust encryption methods, access controls, and secure data storage practices to safeguard the sensitive medical data collected during clinical trials. Ensuring data security is essential to maintain the trust of participants and protect the integrity of research findings [55].

Policy and Ethical Considerations Surrounding 5G Implementation in Healthcare

Patient consent and data sharing: Patient consent is a cornerstone of ethical healthcare practices in the 5G era. Informed consent is essential for the collection and sharing of medical data over 5G networks. Patients must have a clear understanding of how their data will be used, who will have access to it, and for what purposes. They should be able to provide or withhold consent based on this understanding. Ethical healthcare practices prioritize patient autonomy, ensuring that individuals have the right to make informed decisions about their healthcare data [56].

Patient privacy: Protecting patient privacy is a fundamental ethical obligation in 5G-enabled healthcare. Healthcare providers and technology vendors must implement robust privacy measures to keep patient data confidential and secure. This includes encryption to protect data during transmission and storage, access controls to restrict data access to authorized personnel, and data anonymization to reduce the risk of re-identification. These measures align with the ethical principle of respecting patient confidentiality and maintaining trust in healthcare services [57].

Equitable access: Ethical principles emphasize the need for equitable access to 5G-enabled healthcare services. The ethical imperative is to ensure that the benefits of 5G technology reach all segments of the population, regardless of geographic location or socioeconomic status. This involves efforts to bridge the digital divide and make healthcare services accessible to underserved and vulnerable populations. Ethical healthcare practices aim to reduce disparities in healthcare access and outcomes [58].

Data ownership and control: Ethical considerations encompass the ownership and control of healthcare data. Patients should have control over their medical data, including access, management, and even revoking access to their data as they see fit. Data ownership and control empower patients to make decisions about the use and sharing of their healthcare information. These ethical principles uphold the concept of patient autonomy and self-determination [59].

Ethical research practices: Ethical guidelines for research conducted using 5G technology prioritize the safety and well-being of research participants. This includes obtaining informed consent ensuring that research participants are fully aware of the study's purpose and potential risks. Ethical research practices also involve minimizing risks to participants, ensuring transparency in research procedures, and obtaining ethical approval from institutional review boards. Research ethics protect the rights and welfare of individuals involved in scientific studies [60].

Responsible innovation: Responsible innovation is an ethical imperative in the 5G-enabled healthcare landscape. Policymakers, healthcare organizations, and technology providers have a responsibility to innovate while upholding ethical standards. This involves regularly evaluating the ethical implications of new technologies and practices. Ethical considerations should be an integral part of the innovation process, guiding the development and deployment of healthcare technologies in ways that prioritize patient well-being, privacy, and safety [61].

Compliance with regulations: Ethical healthcare practices involve the development and enforcement of regulations that govern the use of 5G technology in healthcare. These regulations should reflect the evolving ethical considerations and the need to protect patient rights and safety. Regulatory compliance ensures that ethical standards are upheld and provides a legal framework for holding individuals and organizations accountable for their actions. Ethical regulations in healthcare are a means of safeguarding patient interests and maintaining the integrity of the healthcare system [62].

Future implications and recommendations

Regulatory Framework and Guidelines for 5G Implementation in Healthcare

Data privacy and security: Data privacy and security are at the core of the regulatory framework for 5G-enabled healthcare. The guidelines should provide detailed instructions on how patient data is collected, shared, stored, and protected to safeguard patient privacy and confidentiality. This includes specifying encryption and access control requirements, as well as data retention and disposal policies. Compliance with established data protection regulations, such as HIPAA in the United States, is imperative. These regulations set the standard for data security in healthcare and serve as a foundation for protecting patient information [63].

Patient consent: The regulatory framework should clearly outline procedures for obtaining informed patient consent in the context of 5G-enabled healthcare services. Patients must be fully aware of how their data will be used, who will have access to it, and for what purposes. They should have the right to make informed choices about data sharing and participation in healthcare programs. This emphasis on patient consent aligns with the principles of patient autonomy and informed decision-making, ensuring patients have control over their healthcare data [64].

Quality of service: The regulatory framework should set stringent standards to ensure the consistently high quality of service in 5G-enabled healthcare. This includes requirements for network reliability, data transfer speeds, and low latency, particularly for applications that demand real-time data transmission. Ensuring a high-quality network infrastructure is essential to support telemedicine, remote surgery, and other critical healthcare services that rely on uninterrupted and real-time data exchanges [65].

Interoperability: The regulatory guidelines should strongly encourage interoperability among 5G-enabled healthcare devices and systems. These guidelines should specify technical standards and data exchange protocols to ensure that healthcare data can flow seamlessly between various devices and platforms. Interoperability is crucial for delivering comprehensive patient care, enabling healthcare professionals to access complete and up-to-date patient information, regardless of the devices or systems used [65].

Innovation promotion: While emphasizing data privacy and security, the regulatory framework should also strike a balance by promoting innovation in healthcare. It should encourage the development and deployment of technologies that enhance patient care and outcomes. Innovations that comply with ethical and privacy standards should be actively supported. This approach fosters a culture of continuous improvement in healthcare and allows for the development of new technologies that can benefit patients and improve healthcare delivery [66].

Compliance monitoring: To ensure the effectiveness of the regulatory framework, regular monitoring and auditing of healthcare providers, technology vendors, and research institutions are essential. These audits should assess compliance with the established regulations and guidelines. Non-compliance should be addressed promptly through corrective actions and penalties if necessary. Compliance monitoring is crucial for maintaining the integrity of the regulatory system and upholding patient rights and data security [67].

Collaboration: Policymakers, healthcare organizations, and technology vendors should collaborate continuously to refine and adapt the regulatory framework as technology and healthcare practices evolve. Collaboration ensures the framework remains relevant and effective in the dynamic healthcare landscape. It allows for incorporating the latest scientific findings, emerging technologies, and evolving ethical standards, ensuring that healthcare regulations keep pace with advancements in 5G technology [68].

Ethical considerations: The regulatory framework should comprehensively address ethical considerations in 5G-enabled healthcare. This includes considerations related to patient data ownership and control, equitable access to healthcare services, and responsible research practices. The framework should uphold ethical principles such as patient autonomy, beneficence, and non-maleficence, ensuring that technology and healthcare practices align with societal values and prioritize patient well-being and rights. Ethical guidelines guide the development and implementation of 5G-enabled healthcare technologies, emphasizing the responsible and ethical use of these technologies [69].

Continued Research on the Health Impacts of 5G Radiation

Long-term health studies: Longitudinal studies are crucial for addressing concerns about the long-term health effects of 5G radiation exposure. These studies should track the health outcomes of individuals exposed to 5G technology over extended periods, potentially spanning several years or even decades. By observing and analyzing health data over time, researchers can identify potential health risks that may emerge with prolonged exposure to 5G radiation. Long-term health studies are vital for providing a comprehensive understanding of the health impact of 5G technology [24].

Risk assessments: Rigorous risk assessments are essential to evaluate the potential health hazards associated with 5G radiation. These assessments should be conducted with meticulous attention to detail, considering various exposure scenarios. This includes examining different frequencies, power levels, and durations of exposure to 5G radiation. By systematically assessing the risks associated with varying conditions of exposure, researchers can provide a nuanced understanding of the potential health effects, helping to improve safety standards and guidelines [26].

Exposure standards: The ongoing research on 5G radiation can contribute to the development and refinement of exposure standards and guidelines. These standards should be based on the latest scientific findings and designed to protect public health while allowing for the beneficial use of 5G technology. As research advances, exposure standards may need to be adjusted to reflect the evolving understanding of the health impact of 5G radiation. Clear and well-defined exposure standards are critical for safeguarding public health [70].

Biological mechanisms: Researchers should investigate the biological mechanisms underlying any potential health effects of 5G radiation. Understanding how 5G radiation interacts with biological tissues and cellular processes is essential for assessing its safety. This research should explore whether 5G radiation has any measurable impact on biological systems, such as DNA damage, oxidative stress, or cellular function. Insights into the biological mechanisms can help determine the plausibility of health risks and inform safety assessments [71].

Transparency and communication: Transparent and clear communication of the results of health impact studies is crucial. It ensures that the public is informed about the potential risks and benefits of 5G technology. Openly sharing research findings and their implications, along with addressing any uncertainties, fosters public trust and empowers individuals to make informed decisions regarding their exposure to 5G technology. Effective communication is a cornerstone of responsible and ethical scientific research [72].

Regulatory decision-making: Regulatory bodies should base their decisions about the deployment and use of 5G technology on the latest scientific research. Continued health impact studies are vital for regulators to make informed decisions and set appropriate safety standards. The research should be a key driver in shaping regulatory policies, ensuring that they align with the current state of scientific knowledge and prioritize public health [2].

Public health guidelines: Public health guidelines should be updated to reflect the evolving understanding of 5G radiation. These guidelines play a significant role in helping individuals and healthcare providers make informed decisions about their exposure to 5G technology. Guidelines should provide practical recommendations for minimizing potential risks while harnessing the benefits of 5G, and these recommendations should be based on the most up-to-date research [2].

International collaboration: Given the global nature of 5G deployment, international collaboration in research is essential. Researchers and regulatory bodies from different countries should work together to ensure a comprehensive understanding of 5G radiation's health impacts. International collaboration allows for the pooling of expertise and resources, promotes data sharing, and ensures that research findings are applicable across diverse geographic regions. A global approach to research enhances the credibility and reliability of findings and helps establish common safety standards [73].

Cybersecurity Protocols and Safeguards for Medical Data

Collaboration and information sharing: Collaboration and information sharing among healthcare institutions, device manufacturers, and network providers are essential components of a robust cybersecurity strategy. By working together and sharing information about emerging threats and best cybersecurity practices, these stakeholders can collectively strengthen their defense against cyberattacks. Collaborative efforts can lead to the early detection and mitigation of threats, as well as the development of effective security protocols that consider the entire healthcare ecosystem [74].

Robust encryption: Robust encryption plays a central role in safeguarding medical data. Encryption should be applied during data storage and transmission to protect patient information from unauthorized access. End-to-end encryption ensures data is secure throughout its journey, while data-at-rest encryption safeguards information stored on devices and servers. This level of encryption ensures that even if data is intercepted or compromised, it remains unintelligible to unauthorized parties [75].

Access controls: Access controls are crucial for limiting access to sensitive patient data and medical devices. Implementing multi-factor authentication and role-based access controls ensures that only authorized personnel can access these resources. This not only protects patient information but also prevents unauthorized individuals from tampering with or manipulating medical devices, which could have life-threatening consequences [76].

Security audits: Regular security audits are proactive measures to identify vulnerabilities and weaknesses in healthcare systems and devices. These audits thoroughly assess the entire cybersecurity infrastructure to pinpoint potential security issues. By conducting security audits, healthcare organizations can address these issues before they are exploited by malicious actors, strengthening the overall security posture [77].

Patch management: Patch management is critical for keeping software and systems up-to-date and secure. Healthcare organizations and device manufacturers should have effective patch management systems to ensure timely software updates. Regular updates help in closing security vulnerabilities and protecting against known threats. Failure to keep systems updated can leave them susceptible to exploitation [78].

Intrusion detection systems: Intrusion detection systems are indispensable for monitoring networks and devices in real-time for suspicious activities. These systems can swiftly identify anomalies, patterns of behavior indicative of cyberattacks, and potential security breaches. By responding to these incidents in real time, intrusion detection systems help minimize the damage caused by cyberattacks and safeguard patient data [47].

Security awareness training: Healthcare staff must receive training to recognize and respond to cybersecurity threats. An educated workforce is a critical component of a strong security posture. Training helps staff understand the risks, identify suspicious activities, and take appropriate actions. Security awareness training empowers healthcare professionals to play an active role in protecting patient data and the healthcare infrastructure [79].

Incident response plans: Healthcare organizations should have well-defined incident response plans. These plans outline how to respond to security breaches, minimize damage, and recover from cyberattacks. A clear and organized response to security incidents ensures the impact is mitigated and patient care remains uninterrupted [80].

Continuous monitoring: Continuous monitoring of security threats and vulnerabilities is essential, given the rapidly evolving threat landscape in healthcare. Healthcare organizations should remain vigilant and proactive in identifying and mitigating risks as they evolve. Continuous monitoring provides early warning of potential threats, allowing for timely responses and preventive measures [37].

Compliance with regulations: Healthcare institutions must adhere to data protection and cybersecurity regulations, such as HIPAA in the United States. Compliance with these standards is not only a legal requirement but also a crucial step in protecting patient data and maintaining the trust of patients. Regulatory compliance ensures that healthcare organizations meet minimum security standards and avoid legal repercussions for data breaches [81].

Training and Education for Healthcare Professionals in 5G Technology

Device operation and usage: Healthcare professionals require comprehensive training in operating and utilizing 5G-enabled medical devices. This training involves gaining a deep understanding of the functionalities, features, and data outputs of these devices. Healthcare professionals should be proficient in device setup, calibration, maintenance, and troubleshooting to ensure the accurate and safe use of 5G-enabled medical technology. Proficiency in device operation is crucial to harnessing the benefits of 5G technology effectively [2].

Data interpretation: Healthcare professionals need the skills to interpret and analyze the data generated by 5G-enabled devices. This training includes understanding how to use real-time data for various aspects of patient care, such as continuous monitoring, diagnostics, and treatment decision-making. Healthcare professionals should be able to extract meaningful insights from data streams and apply them to enhance patient outcomes, making data interpretation a core competency in the 5G era [17].

Privacy and ethical considerations: Education on the ethical and privacy standards of 5G technology in healthcare is vital. Healthcare providers should know the ethical principles and legal requirements for patient consent, data security, and confidentiality. This training ensures that healthcare professionals prioritize patient privacy, data protection, and ethical decision-making [63].

Cybersecurity awareness: Given the critical importance of safeguarding patient data, healthcare professionals should be well-versed in recognizing and responding to cybersecurity threats. Training in this area includes understanding how to identify potential security breaches, how to report security incidents, and how to take immediate action to mitigate risks. Cybersecurity awareness is essential to protect patient data and maintain the integrity of healthcare systems [82].

Compliance with regulations: Training should cover the regulatory standards and guidelines relevant to 5G technology in healthcare, such as HIPAA compliance in the United States. Healthcare professionals should be well-informed about these regulations to ensure their practices align with legal requirements. Compliance not only helps protect patient data but also mitigates the risk of legal repercussions [7].

Interdisciplinary collaboration: The complexities of 5G technology in healthcare necessitate training healthcare professionals to collaborate effectively with IT experts and cybersecurity specialists. This interdisciplinary collaboration is vital to address the multifaceted challenges that arise in the integration of 5G technology. Effective teamwork between healthcare and technology experts ensures that healthcare services are delivered securely and efficiently [2].

Continuous learning: In the rapidly evolving landscape of technology and healthcare practices, training should be a continuous process. Healthcare professionals should engage in lifelong learning to stay updated on the latest advancements and best practices related to 5G technology. Continuous learning ensures that professionals remain competent and adapt to the changing demands of the healthcare industry [2].

Simulation and hands-on training: Practical training through simulations and hands-on experience with 5G-enabled medical devices is invaluable. This type of training allows healthcare professionals to apply their knowledge in real-world scenarios, enhancing their confidence and competence in using these devices. Simulations also provide a safe environment to practice critical skills and decision-making [83].

Public awareness and communication: Effective communication with patients about using 5G technology and its potential benefits is essential. Healthcare professionals should receive training on conveying complex technological information in a way that patients can understand. Building patient trust through transparent communication is crucial and helps ensure informed consent for using 5G-enabled healthcare services [2].

Professional certifications: Certification programs specific to 5G technology in healthcare can serve as valuable credentials to validate the skills and knowledge of healthcare professionals. These certifications can demonstrate a healthcare professional's competence in using 5G technology and adherence to best practices in this rapidly evolving field, instilling confidence in patients and employers [2].

Ethical Standards for the Use of 5G in Medicine

Informed patient consent: In the context of 5G technology in healthcare, informed patient consent is a foundational ethical principle. It means that before collecting and sharing their medical data using 5G technology, patients should be provided with comprehensive information about the purpose, nature, and potential implications of such data usage. Patients must be fully aware of how their data will be used, who will have access to it, and what the potential benefits and risks are. This empowers patients to make autonomous decisions about their healthcare data, ensuring their rights and preferences are respected [84].

Data ownership and control: This ethical standard revolves around the concept that patients should have ownership and control over their medical data. In practical terms, this means that patients should be able to access their healthcare information, manage it (e.g., correct inaccuracies), and, if they choose, revoke access to specific individuals or entities. It recognizes that patients have a vested interest in the confidentiality and integrity of their medical data, reinforcing the principle of patient autonomy in healthcare decision-making [83].

Data privacy and security: Data privacy and security are integral to ethical healthcare practices. Ethical standards should prioritize the robust protection of patient data against breaches and unauthorized access. This involves implementing encryption, access controls, and other security measures to ensure the confidentiality and integrity of healthcare information. It reflects the ethical duty to safeguard patient privacy and maintain the trust and confidence of individuals in the healthcare system [85].

Equitable access: Ethical standards should address the ethical imperative of equitable access to 5G-enabled healthcare services. This principle asserts that all individuals, regardless of their geographic location or socioeconomic status, should have access to the benefits of 5G technology in healthcare. It embodies the ethical concept of distributive justice, striving to ensure that healthcare disparities are minimized and that healthcare resources are distributed fairly [86].

Beneficence and non-maleficence: These principles are fundamental to ethical medical practices. Beneficence emphasizes acting in the best interest of the patient, striving to maximize their well-being and health outcomes. Non-maleficence, on the other hand, focuses on the obligation to "not harm." In the context of 5G technology in healthcare, these principles guide decisions and actions to ensure patients receive the most beneficial and least harmful care, prioritizing patient safety and health [87].

Transparency and informed decision-making: Ethical standards require transparency in healthcare practices and communication. Patients should be provided with clear, honest, and understandable information about the potential implications, risks, and benefits of using 5G technology in their healthcare. This empowers patients to make informed decisions about their care, respecting their autonomy and right to participate actively in their healthcare decisions [2].

Professional ethics: Healthcare professionals are held to ethical standards of professional conduct. This includes respecting patient confidentiality, providing high-quality care, and upholding principles such as patient autonomy. Ethical standards reinforce the importance of maintaining the trust of patients and the ethical obligations of healthcare professionals in their practice [88].

Ethical research practices: When research involves the use of 5G technology in healthcare, ethical guidelines are essential. These guidelines include obtaining informed consent from research participants, minimizing research-related risks, and ensuring the responsible conduct of research. Ethical research practices prioritize the well-being and rights of research participants and the integrity of the research process [89].

International ethical collaboration: Given the global nature of 5G technology and healthcare, international collaboration on ethical standards and guidelines is vital. This collaboration helps ensure a consistent and ethical approach to using 5G in healthcare, particularly when research, data sharing, or healthcare services span international boundaries [7].

Continuous ethical evaluation: Ethical standards should be dynamic and subject to continuous evaluation and adaptation. In a rapidly evolving healthcare and technological landscape, ongoing ethical assessments are essential to ensure that standards remain relevant, ethical issues are addressed in real time, and the highest ethical standards are upheld in healthcare practices. This reflects the commitment to adapt and improve ethical standards as technology and healthcare practices evolve [90].

## Conclusions

In conclusion, the integration of 5G technology in the medical sector brings forth a range of benefits, including the potential for enhanced telemedicine services, accelerated data transfer for medical records, improved remote surgery capabilities, real-time monitoring and diagnostics, advancements in wearable medical devices, and the promise of precision medicine. However, it also introduces a set of risks, such as concerns about radiation exposure, cybersecurity vulnerabilities, technology dependence, and privacy issues. Striking a balanced approach is paramount, as the responsible implementation of 5G in healthcare requires stringent regulatory oversight, ethical considerations, and ongoing research to ensure its safe and efficient use. As we envision the future of 5G in the medical field, it is evident that this technology can revolutionize healthcare by enhancing patient care, broadening access to medical expertise, and personalizing treatments. To achieve this future, we must navigate the landscape with a comprehensive understanding of its potential and challenges, ensuring the responsible and ethical use of 5G technology in medicine.
